# Temporal Retinal Nerve Fiber Loss in Patients with Spinocerebellar Ataxia Type 1

**DOI:** 10.1371/journal.pone.0023024

**Published:** 2011-07-29

**Authors:** Sarah Stricker, Timm Oberwahrenbrock, Hanna Zimmermann, Jan Schroeter, Matthias Endres, Alexander U. Brandt, Friedemann Paul

**Affiliations:** 1 Klinik für Neurologie, Charité – Universitätsmedizin Berlin, Berlin, Germany; 2 NeuroCure Clinical Research Center and Experimental and Clinical Research Center, Charité – Universitätsmedizin Berlin and Max Delbrueck Center for Molecular Medicine, Berlin, Germany; 3 Klinik für Augenheilkunde, Charité – Universitätsmedizin Berlin, Berlin, Germany; Institute Biomedical Research August Pi Sunyer (IDIBAPS) - Hospital Clinic of Barcelona, Spain

## Abstract

**Background:**

Autosomal dominant spinocerebellar ataxia type 1 is an adult onset progressive disorder with well characterized neurodegeneration in the cerebellum and brainstem. Beyond brain atrophy, few data exist concerning retinal and optic nerve involvement.

**Objective:**

To evaluate retinal changes in SCA1 patients compared to age and gender matched healthy controls.

**Methodology/Principal Findings:**

Nine patients with SCA1 were prospectively recruited from the ataxia clinic and were compared to nine age and gender matched healthy controls. Both cohorts received assessment of visually evoked potentials and eye examination by optical coherence tomography to determine retinal nerve fiber layer thickness and total macular volume. While no differences were found in visually evoked potentials, SCA1 patients showed a significant reduction of mean retinal nerve fiber layer thickness (RNFLT) compared to healthy controls (84±13 µm vs. 97±8 µm, p = 0.004). Temporal areas showed the most prominent RNFLT reduction with high statistical significances (temporal-inferior: p<0.001, temporal: p<0.001, temporal-superior: p = 0.005) whereas RNFLT in nasal areas was in the range of the control group. From six SCA1 patients an additional macular scan was obtained. The comparison to the corresponding healthy control showed a slight but not significant reduction in TMV (8.22±0.68 mm^3^ vs. 8.61±0.41 mm^3^, p = 0.15).

**Conclusion:**

In SCA1 patients, we found evidence for degeneration of retinal nerve fibers. The temporal focus of the observed retinal nerve fiber layer reduction suggests an involvement of the papillo-macular bundle which resembles pathology found in toxic or mitochondrial optic nerve disease such as Leber's hereditary optic neuropathy (LHON) or dominant optic atrophy (DOA).

## Introduction

Spinocerebellar ataxia type 1 (SCA1) is an autosomal-dominantly inherited, late-onset neurodegenerative disease primarily affecting the cerebellar cortex and brainstem. Affected patients suffer from disturbed motor coordination, slurred speech, dysphagia, spasticity, extrapyramidal movements such as dystonia or chorea, cerebellar oculomotor disturbances and ophthalmoparesis, saccade slowing and - at late disease stage - cognitive impairment [1]. A gain-of-function toxicity of the ataxin 1 protein was identified as the main causative agent in SCA1: In the ATXN1 gene on chromosome 6p23 a CAG-repeat expansion of variable length reaching between 39 and 83 repeats encodes for a prolonged polyglutamine chain in ataxin1 protein [2]. The physiologic function of ataxin1 is barely understood [3]. Subsequent nuclear and cytosolic protein aggregation in cerebellar Purkinje cells and brainstem neurons finally leads to cell death. Patients with longer CAG repeat tend to have an earlier disease onset and a faster progression than patients with short repeat expansions, causing great variations in disease severity even within families [1].

Historically the classification of spinocerebellar ataxias has been directed by additional extracerebellar sign presence (autosomal dominant ataxia type I, ADCA I) or absence (ADCAIII), with cerebellar ataxia plus retinal degeneration being termed ADCA II [4]. Since genetic testing became available, at least 30 spinocerebellar ataxia subtypes have been identified with different gene localisation. SCA1 belongs to ADCA I group without known retinal involvement, whereas in SCA7 (ADCA II) degeneration of macula or retina is well established [5] [6]. In SCA1 patients, however, some groups found ophthalmologic pathology besides well described cerebellar oculomotor abnormalities: Visual acuity reduction with color vision failure and visual field contraction are reported. Some case collections suggest an increase of these symptoms with disease duration [7] [8], but do not find prominent changes at disease onset [8]. Electroretinogram (ERG) is reported to show mild attenuation of oscillatory potentials and corneal endothelial cell density was reported decreased [8]. The reduced visual acuity in SCA1 has been attributed to optic atrophy, which was first described by fundus examination [8]. Optic nerve involvement was also suggested by a significant percentage of VEP abnormalities in SCA1 patients [9] [10]. However in these studies 40–50% of SCA1 patients did not show VEP alteration and earlier neuro-pathologic work could not establish retinal or optic nerve pathology in SCA1 [11]. In summary, descriptions on retinal and optic nerve changes in SCA1 are controversial and no data exists on retinal pathology using high resolution OCT so far.

Optical coherence tomography (OCT) is a modern non-invasive method for high-resolution retinal investigations. The recently developed spectral domain technique allows spatial resolution down to 3 µm when measuring retinal nerve fiber layer thickness (RNFLT), which has previously only been possible with histopathology and ranges far above MRI resolution [12]. OCT has become a powerful tool in ophthalmologic diagnostics of diseases affecting retinal tissue e.g. glaucoma [12] or multiple sclerosis [13] [14]. Since it allows unique morphologic characterization of neuronal cells belonging to the CNS, the method is currently explored for diagnosis and monitoring of disease progression in a variety of neurodegenerative disorders as a surrogate parameter for cerebral and/or optic nerve axonal loss [15] [16] [17].

Due to its simple application even in severely handicapped individuals the method is well suited for examination of ataxia patients. It has so far been applied in SCA7, where a significant reduction in RNFLT in all quadrants sparing the temporal one is consistently found [6] [5]. To the best of our knowledge in other forms of SCA no systematic study of retinal involvement using OCT has been performed so far.

Against this background, we set out to examine individuals with genetically proven SCA1 compared to age-and-gender matched healthy controls with OCT and VEP for retinal and optic nerve involvement.

## Methods

### Ethics statement

The study was approved by the institutional ethics committee and all participants gave informed written consent.

### Objectives

To evaluate retinal changes in SCA1 patients compared to age and gender matched healthy controls.

### Participants

Patients were recruited from the ataxia outpatient clinic of the Charité university hospital in Berlin from December 2009 to January 2011. Inclusion criteria were a genetically diagnosed SCA1 and age ranging from 18 to 75 years. Healthy controls were matched by gender and age with a tolerance of ±3 years. Participants with known pre-existing ophthalmological diseases were excluded.

If available, patients provided data of the number of CAG repeats in the ATXN1 gene, which were genetically ascertained by external laboratories. The disease duration was defined as the time (in years) since the first symptom appeared. Visual Acuity (VA) was assessed using Snellen Charts and refractive error (REF) was estimated using the Heidelberg Spectralis integrated confocal scanning laser ophthalmoscope (SLO).

### Clinical examination

The SARA score was used for assessment and rating of patients' ataxia [18]. Briefly, the test assesses the following functions: gait, stance, sitting, speech disturbance, finger chase, nose-finger test, fast alternating hand movements and heel-shin slide. A summary score of these functional items gives the final SARA score, with a range from 0 (no ataxia) to 40 (most severe ataxia).

### Optical Coherence Tomography

**Figure 1:**
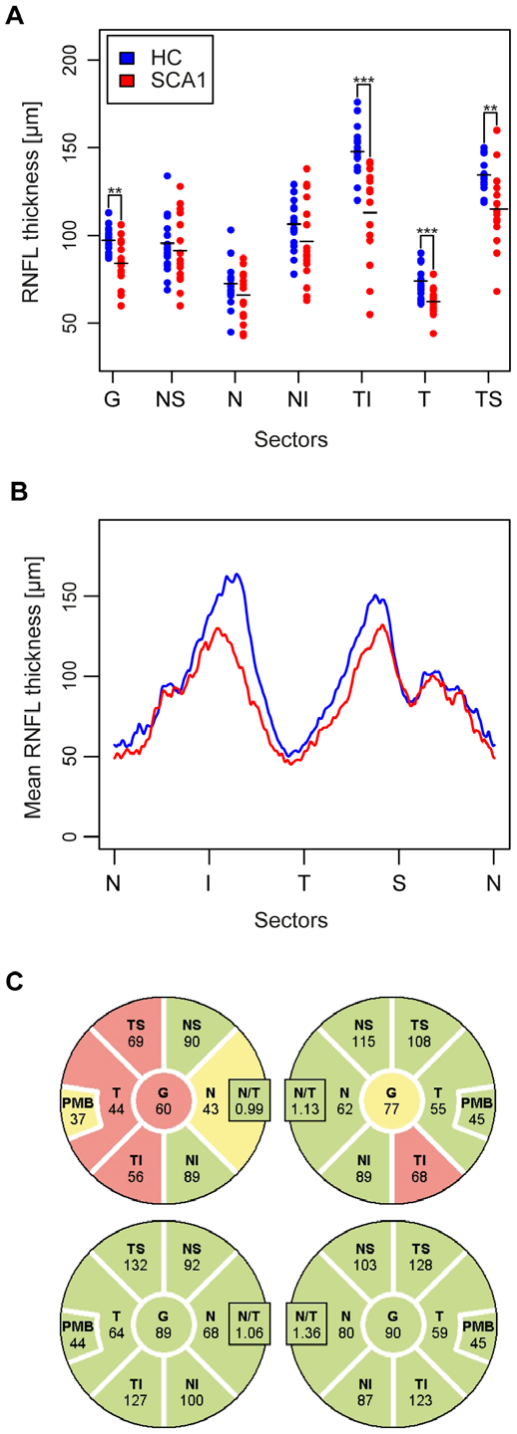
Differences of RNFLT between SCA1 and HC. (A) Average RNFLT (in µm) of the total ring scan (G) and in the different sectors (nasal-superior (NS), nasal (N), nasal-inferior (NI), temporal-inferior (TI), temporal (T) and temporal-superior (TS)). Error bars indicate standard deviation. Significance levels (*** for p<0.001, ** for p<0.01 and * for p<0.05) were calculated by GEE. (B) Mean RNFLT profile of both study groups in µm. (C) Both eyes (OD = right, OS = left) of a sample patient (upper line) with typical RNFLT sectors in comparison with matched healthy control (bottom line). The colors of the sectors are related to the comparison with the Spectralis normative database. Green indicates thickness values within the 95%-percentile of the database, while sectors in yellow were thinner than the 5%-percentile of the normative database and sectors in red were within the lowest 1%.

A spectral domain OCT device (Heidelberg Spectralis SD-OCT, Heidelberg Engineering, Germany, Spectralis software version 5.2.4.0, Eye Explorer Software 1.6.2.0) was used to measure the RNFLT and the total macular volume (TMV). For RNFLT, three 3.4 mm circular scans were acquired using the standard protocol and the scan with the highest quality value was chosen (in case of equal quality, an arbitrary scan was taken). The thickness between the inner limiting membrane and the retinal nerve fiber layer was calculated by the device software's segmentation algorithm. As parameters we used the average RNFLT of the full circle scan (G) and the partition of the circle in the following sectors: nasal-superior (NS), nasal (N), nasal-inferior (NI), temporal-inferior (TI), temporal (T) and temporal-superior (TS). The partition is visualized in figure 1C.

**Figure 2:**
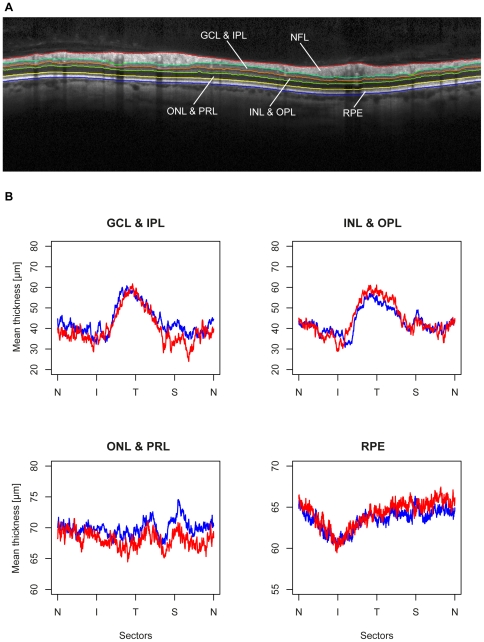
Segmentation of retinal layers. (A) Sample ring scan with additional layer segmentation. Layers from top: Nerve fiber layer (NFL), ganglion cell layer (GCL), inner plexiform layer (IPL), inner nuclear layer (INL), outer plexiform layer (OPL), outer nuclear layer (ONL), photoreceptor layer (PRL) and retinal pigment epithelium (RPE). (B) Comparison of the mean thickness of the additional retinal layers between healthy controls (in blue) and SCA1 patients (in red).

For the analysis of further retinal tissue layers, the program OCTseg [19] was used for automatic layer segmentation. Each segmentation result was manually corrected in a blinded fashion and the exported segmentation lines were used to calculate the thicknesses of the following layers: ganglion cell layer (GCL) and inner plexiform layer (IPL), inner nuclear layer (INL) and outer plexiform layer (OPL), outer nuclear layer (ONL) and photoreceptor layer (PRL) and the retinal pigment epithelium (RPE) (figure 2A). The RNFL segmentation of OCTseg confirmed the previous results.

The TMV was appointed by a custom protocol which generated 61 slices (B-scans) focusing the fovea centralis with a scanning angle of 30°×25° and a resolution of 768 A-scans per B-scan. Some patients were not able to focus the fixation point during examination due to strong eye and head movement. TMV was calculated by estimating the distance between the inner limiting membrane and the Bruch-membrane in a cylinder with 6mm in diameter using the device software's segmentation algorithm.

All measurements were performed by one of three experienced operators. The computationally generated segmentation lines were reviewed by the operators and manually corrected in cases of errors.

### Visually Evoked Potentials

VEPs were recorded from Oz electrode against a Cz reference electrode following checkerboard stimulation. Between 40 and 80 recordings were averaged twice until clear peaks became visible. The latencies of the P100 peaks were used for analysis. Responses of P100 above 119 ms were defined as abnormal.

### Statistical methods

A Mann-Whitney U-test was used to identify statistical differences of age between the study cohorts. Spearman's Rho tests were used for cross-correlation analysis of the clinical parameters (SARA score, disease duration and number of repeats) within the SCA1 cohort.

Statistical differences between SCA1 patients and the group of healthy controls were calculated using General Estimation Equation (GEE) models to account for within-subject inter-eye dependencies. The multiple tests of all sectors from all retinal layers were adjusted using Bonferroni-Holm Correction. Correlation analysis of a clinical parameter (SARA score, disease duration and visual acuity) to the OCT data (RNFLT and macular volumes) were equally performed with GEE. In all GEEs, OCT measurements were included as the dependent variable.

All statistical analyses and graphical representations were performed with R (R version 2.12.1) including the packages geepack for calculating GEE, psych, Hmisc for basic statistics and plotrix and gplots for graphic generation. All results from R were validated using SPSS 18 (SPSS, Chicago, IL, USA). Graphical presentations were prepared using Adobe Illustrator CS5 (Adobe Systems, Munich, Germany).

## Results

### Cohort Description

In this study, we included nine patients with a genetically diagnosed SCA1 and compared them to a gender- and age-matched cohort of nine healthy controls. Demographic parameters of the investigated cohorts and the clinical results of the SARA score, VEP, visual acuity, refractive error and the number of repeats in the polyglutamine trace of the ATXN1 alleles are summarized in table 1. Due to the small sample size and the pilot character of this study, an additional overview with single patient profiles is given in table 2. The difference in age between the groups was not significant (Mann-Whitney U test, p = 0.93).

**Table 1:** Demographic information and clinical of the patient cohort (SCA1) and the matched healthy controls group (HC).

		SCA1	HC
**Subjects**	N	9	9
**Gender**	Male (%)	5 (56%)	5 (56%)
	Female (%)	4 (44%)	4 (44%)
**Age**	Mean (SD)	51.7 (8.8)	50.7 (8.7)
	Range	39.0 – 67.0	38.0 – 66.0
**Disease duration [in years]**	Mean (SD)	13.1 (8.3)	N/A
	Range	3.0 – 30.0	N/A
**SARA score**	Median (SD)	15.0 (7.4)	N/A
	Range	5.0 – 28.0	N/A
**CAG repeats (diseased allele) [number of repeats]**	Mean (SD)	48.1 (2.9)	N/A
	Range	45 – 53	N/A
**CAG repeats (healthy allele) [number of repeats]**	Mean (SD)	30.7 (1.8)	N/A
	Range	28 – 33	N/A
**Refractive error [D]**	Mean (SD)	−0.68 (1.33)	0.19 (1.50)
	Range	−2.90 – 1.84	−1.67 – 3.95
**Visual acuity [decimal]**	Mean (SD)	0.54 (0.32)	0.86 (0.17)
	Range	0.13 – 1.20	0.62 – 1.11
**VEP P100 latency [ms]**	Mean (SD)	102.9 (5.6)	102.4 (3.3)
	Range	94.3 – 111.0	98.0 – 109.0

Results for the VEP examination were available for 6 SCA1 patients and of 5 of the matching healthy controls. For 1 SCA1 patients repeat length of the diseased allele were missing and 2 patients lack values for the healthy allele and 1 patient had no examination of the visual acuity.

**Table 2:** Single case reports of SCA1 patients.

Case No.	Clinical findings			VEP (OD,OS)	OCT examination (OD, OS)							
	SARA score	Disease duration	Repeats (diseased allele)	P100	G	NS	N	NI	TI	T	TS	TMV
**1**	5	3	50	104.3, 98.0	83, 85	82, 106	55, 72	93, 106	119, 100	69, 57	118, 112	8.28, 8.25
**2**	28	30	N/A	106.8, 111.3	66, 68	60, 86	43, 49	63, 65	106, 83	62, 58	90, 97	7.11, 6.97
**3**	11.5	4	N/A	N/A, N/A	N/A, 80	N/A, 67	N/A, 72	N/A, 84	N/A, 119	N/A, 62	N/A, 105	8.90, 8.76
**4**	26	16	47	N/A, N/A	60, 77	90, 115	44, 62	88, 89	55, 68	44, 55	68, 108	N/A, N/A
**5**	21	12	53	N/A, N/A	87, 84	84, 78	72, 74	86, 91	125, 125	69, 58	118, 114	N/A, N/A
**6**	13	19	49	99.3, 99.3	96, 97	86, 118	87, 84	122, 129	142, 138	64, 59	114, 109	8.95, 8.99
**7**	15	8	48	109.3, N/A	92, N/A	97, N/A	78, N/A	128, N/A	97, N/A	61, N/A	131, N/A	7.69, N/A
**8**	18	10	45	108.0, 103.8	106, 101	128, 113	77, 70	112, 138	141, 133	78, 70	160, 146	8.46, 8.48
**9**	12.5	16	45	97.5, 94.3	83, 79	76, 75	61, 54	80, 70	131, 126	65, 66	127, 123	8.63, 8.64

**Figure 3:**
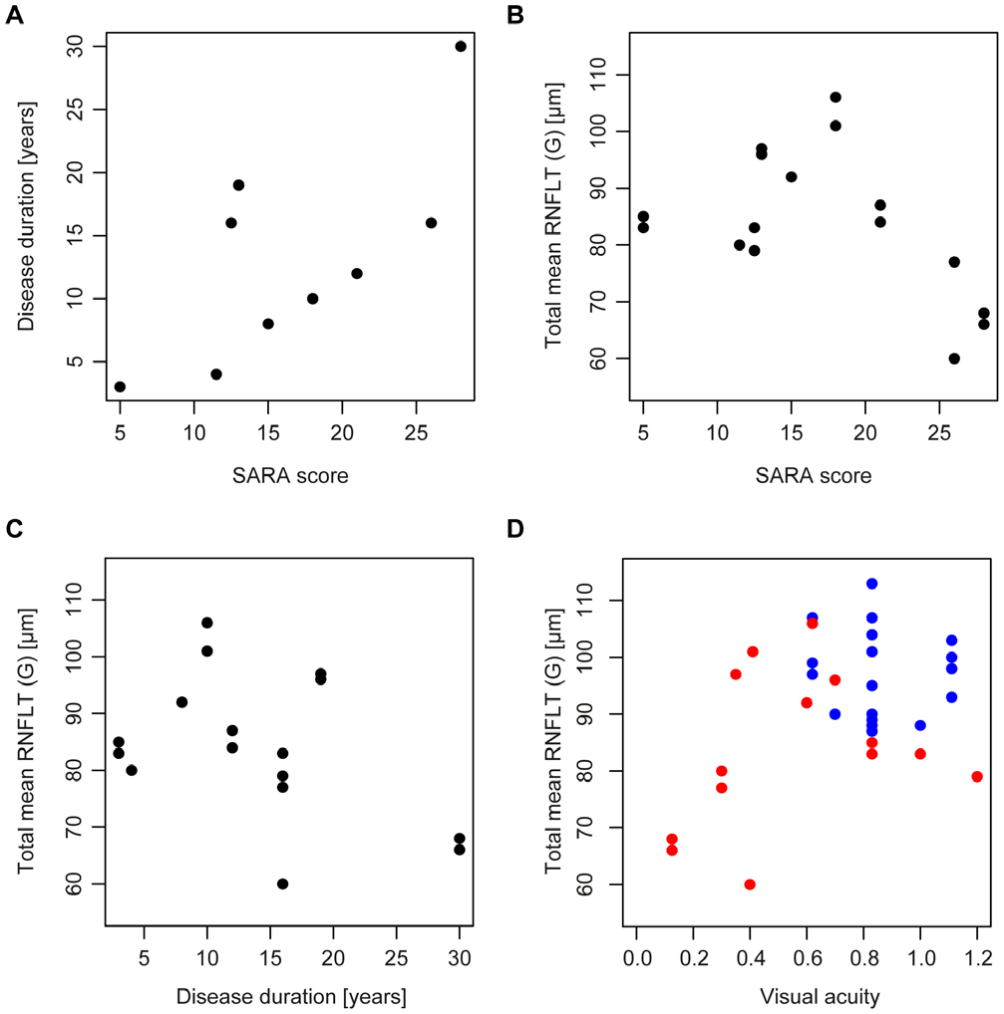
Correlation of clinical parameters with RNFLT. (A) Dependency of disease duration to SARA score. RNFLT with respect to SARA score (B), disease duration (C) and visual acuity (D) for SCA1 patients (red) and healthy controls (blue).

SARA scores for SCA1 patients ranged from 5 to 28 with a median of 15 and the mean disease duration was 13.1 years, ranging from 3 to 30 years. A general tendency towards higher SARA scores for longer disease duration was visible (figure 3A) but did not reach a significant level for the correlation (Spearman's Rho = 0.64, p = 0.061). Seven SCA1 patients provided trinucleotide repeat numbers of the glutamine encoding part of the diseased ATXN1 allele (mean = 48.1, SD = 2.9) and, in addition, six of the patients could provide the repeat numbers of the unaffected allele (mean = 30.7, SD = 1.8).

Refractive error of patients (mean = −0.68D, SD = 1.33D, Range = −2.90 – 1.84D) and healthy controls (mean = 0.19D, SD = 1.50D, Range = −1.67 – 3.95D) did not differ significantly (GEE: p = 0.20). One patient and the corresponding healthy control had to be excluded due to high refractive error.

The visual acuity of SCA1 patients (mean = 0.54, SD = 0.32) was significantly reduced (GEE: p = 0.003) compared to healthy controls (0.86, SD = 0.17).

### Optical coherence tomography

All study subjects (n = 18) underwent an OCT examination for both eyes (36 eyes). For RNFLT, two eyes from SCA1 patients were excluded from further analysis: one right eye due to bad image quality and one left eye due to blindness (post operation of eye tumor). RNFLT was separated as depicted in figure 1C. The total average RNFLT (G) of SCA1 patients was significantly reduced compared to healthy controls (84.0±12.7 µm vs. 97.2±7.7 µm, GEE: p = 0.004). RNFL thinning was predominant and significant in temporal sectors (TS: p = 0.005, T: p<0.001, TI: p<0.001) and was not found in nasal sectors (NS: p = 0.480, N: p = 0.207, NI: p = 0.285) (figure 1A). The most pronounced thinning was found in the temporal-inferior and temporal sectors. When comparing the complete RNFLT profile of both study cohorts (figure 1B), the regional degeneration of the temporal retinal nerve fiber layer became apparent.

The Spectralis device provided a normative database that was used to compare the individual results of the RNFLT sectors to a large cohort of healthy controls. RNFL thickness below 5% of the normative database controls was colored in yellow, while sectors in red indicated values in the 1%-percentile. The normal range of RNFLT values was defined as the 95%-percentile and was colored in green (figure 1C gives an example). Two patients did not have any sectors below the 5%-border while the rest exhibited at least one sector below the normal range. Three patients showed RNFLT values below 1% of the normative database for one or more sectors. Table 3 summarized the occurrence of classifications for the different sectors. In contrast, only a single sector of one healthy control was in the 5%-percentile.

**Table 3:** Comparison of RNFLT results to the Spectralis normative Database.

		SCA1	HC
**Total average RNFLT (G) [µm]**	N eyes classified <5%	6	0
	N eyes classified <1%	3	0
**RNFLT Nasal-Superior (NS) [µm]**	N eyes classified <5%	2	1
	N eyes classified <1%	1	0
**RNFLT Nasal (N) [µm]**	N eyes classified <5%	2	0
	N eyes classified <1%	0	0
**RNFLT Nasal-Inferior (NI) [µm]**	N eyes classified <5%	2	0
	N eyes classified <1%	0	0
**RNFLT Temporal-Inferior (TI) [µm]**	N eyes classified <5%	6	0
	N eyes classified <1%	4	0
**RNFLT Temporal (T) [µm]**	N eyes classified <5%	2	0
	N eyes classified <1%	1	0
**RNFLT Temporal-Superior (TS) [µm]**	N eyes classified <5%	4	0
	N eyes classified <1%	2	0

The mean thickness profiles of the additional retinal layers for both study cohorts are shown in figure 2B. None of the additional layers revealed apparent differences between the groups. When averaging all layer profiles in sectors (data not shown), only the thickness of the NS sector of the ganglion cell and inner plexiform layer showed a reduction in SCA1 patients (GEE: p = 0.001).

Some patients with a distinct nystagmus or uncontrolled head movements were not able to complete the more demanding macular volume scans. Therefore, only 11 eyes of the SCA1 cohort could be analyzed. One TMV measurement of a healthy control had to be excluded due to an erroneous scan. Thus, only the related subjects from the matched group of healthy controls were considered for the cohort comparison. SCA1 patients showed a not significant reduction of the TMV (SCA1: mean = 8.2 mm^3^ (SD = 0.7 mm^3^) vs. HC: mean = 8.6 mm^3^ (SD = 0.4 mm^3^), GEE: p = 0.15) (table 4).

**Table 4:** OCT examination results.

		SCA1	HC	GEE		
				P value	Estimate	Std. err.
**Total average RNFLT (G) [µm]**	Mean (SD)	84.0 (12.7)	97.2 (7.7)	0.00386	−13.0	4.50
	Range	60.0–106.0	87.0–113.0			
**RNFLT Nasal-Superior (NS) [µm]**	Mean (SD)	91.3 (19.6)	95.6 (18.0)	0.480	−4.5	6.32
	Range	60.0–128.0	69.0–134.0			
**RNFLT Nasal (N) [µm]**	Median (SD)	65.9 (13.7)	72.4 (13.0)	0.207	−6.37	5.04
	Range	43.0–87.0	45.0–103.0			
**RNFLT Nasal-Inferior (NI) [µm]**	Mean (SD)	96.5 (23.5)	106.4 (14.4)	0.285	−9.20	8.60
	Range	63.0–138.0	78.0–129.0			
**RNFLT Temporal-Inferior (TI) [µm]**	Mean (SD)	113.0 (26.1)	147.9 (13.7)	0.00007	−35.4	8.86
	Range	55.0–142.0	120.0–176.0			
**RNFLT Temporal (T) [µm]**	Median (SD)	62.3 (7.7)	74.1 (9.2)	0.00072	−11.8	3.48
	Range	44.0–78.0	61.0–90.0			
**RNFLT Temporal-Superior (TS) [µm]**	Mean (SD)	115.0 (21.3)	134.5 (9.9)	0.0052	−19.8	6.90
	Range	68.0–160.0	119.0–150.0			
**Total macular volume (TMV) [mm^3^]**	Mean (SD)	8.2 (0.7)	8.6 (0.4)	0.146	−0.458	0.315
	Range	7.0 – 9.0	8.0–9.1			

RNFLT results were obtained from 9 SCA1 and compared to 9 age/sex matched healthy controls (HC). 2 eyes of SCA1 patients had to be excluded, one left eye due to blindness and one right eye because of errors in the OCT image. In total, 11 eyes of SCA1 patients fulfilled quality criteria for the TMV scan and were compared to the corresponding eyes of the matching healthy controls.

### VEP

For 11 eyes from 6 SCA1 patients VEPs could be acquired in sufficient quality and compared to VEPs of the corresponding healthy controls. None of the participants showed a pathologic increase in latency of the P100 peak and no significant difference between the cohorts was detected (SCA1: mean = 102.9 ms (SD = 5.6 ms) vs. HC: mean = 102.4 ms (SD = 3.3 ms), GEE: p = 0.69).

### Correlation of RNFLT with disease duration,SARA score and visual acuity

Next, OCT findings were correlated with disease duration and disease severity measured by SARA score. When comparing RNFLT (G) with SARA score and disease duration, GEEs for both were not significant (SARA: p = 0.073, duration: p = 0.098). Scatter plots of RNFLT with SARA score and disease duration are given in figures 3B and 3C, respectively, proposing a possible trend for a correlation of RNFLT with SARA score.

Figure 3D show the correlation of the visual acuity with RNFLT (G) for SCA1 patients (red) and healthy controls (blue) with no significant correlations between the combined cohorts (GEE: p = 0.46).

## Discussion

This is the first study investigating retinal involvement in SCA1 patients using optical coherence tomography. We found a pattern of temporal atrophy of the retinal nerve fiber layer in most patients. Layer segmentation analysis confirmed temporal RNFLT reduction whereas no significant differences were seen in average of ganglion cell layer, inner and outer plexiform layer, photoreceptor layer and pigment epithelium. In sector analysis of layers only the NS sector of the ganglion cell and inner plexiform layer showed a reduction in SCA1 patients. Two patients (table 2, cases 6 and 8) displayed no significant RNFLT changes. The patient with longest disease duration (30 years; table 2, case no. 2) and highest SARA score (28) showed additional severe macular atrophy. However, in this patient RNFLT reduction was very prominent, indicating that in late stage disease nerve fiber atrophy could lead to ganglion cell degeneration and consecutively to macular volume reduction. Although a reduction of retinal nerve fiber layer thickness was not present in each individual SCA1 patient, a correlation trend of RNFLT with disease duration and severity was seen. In contrast to OCT, VEP P100 latencies were not able to detect changes in our patients. However, since VEP amplitudes correlate better with axonal loss but were not analyzable in our small sample size due to high inter- and intra- subject variability, we might have missed a significant difference, which might be shown in a larger cohort.

Strengths of the study are the prospective design, the matched control group and the use of spectral domain OCT with layer segmentation analysis. The used OCT device provides an eye tracking function that registers OCT scans to a fundus image and thereby reduces the effects of eye movement during scans. This function makes the application of OCT examination and especially of 3D volume scans possible for patients with severe head ataxia or eye movement abnormalities. However, in several patients this correction was not sufficient, leading to missing scans in the demanding macular volume scans. Another weakness of the study is the low sample size which is owed to the rarity of the disease and the severe disability of many patients. Most likely this contributed to not significant changes to macular volumes and correlations to SARA score or disease duration.

Interestingly, the consistent pattern of selective temporal RNFLT reduction in our study prompts the question of pathophysiologic relevance. One explanation could be that RNFLT analysis enables early detection of incipient optic atrophy in SCA1 since a temporal emphasis of RNFLT reduction is found in a variety of diseases associated with optic nerve atrophy. These conditions comprise post neuritic optic atrophy in multiple sclerosis [16] [20], hereditary optic atrophies such as dominant optic atrophy (DOA) and Leber's hereditary optic neuropathy (LHON) and toxic optic atrophies. However VEP did not detect optic atrophy in our study with all the drawbacks discussed above. But also previous studies using VEP did not find optic nerve involvement to such an extent as our results would suggest [9] [10].

Selective temporal RNFLT involvement could be explained pathophysiologically by a differential vulnerability of retinal axons to mutated ataxin1.The temporal quadrant of the retinal nerve fiber layer is built primarily by parvo-cellular axons from the papillo-macular bundle. These fibers consist of smaller, thinly myelinated axons with rapid firing rates which serve the visual functions of high resolution visual acuity, color vision and high spatial frequency of contrast sensitivity. On the other hand, magno-cellular ganglion cell axons and photoreceptors which are both distributed evenly throughout the retina seem relatively unaffected from damage. Magno-cellular ganglion cells have thicker axons with lower firing rate and serve low-spatial-frequency contrast sensitivity and motion stereopsis.

Other diseases with primary involvement of parvocellular axons comprise the so called non-syndromic mitochondrial optic neuropathies such as Leber's hereditary optic neuropathy (LHON) and OPA1 related dominant optic nerve atrophy (DOA) and toxic and nutritional optic neuropathies such as tobacco-alcohol amblyopia or ethambutol-induced optic neuropathy [21].

As common etiologic pathway of such disparate diseases the disruption of mechanisms participating in the correction of oxidative stress with primary insult to mitochondria is discussed [21] [22]. Due to their small volume and fast firing response, parvo-cellular axons may be more susceptible to energy depletion by defective oxidative phosphorylation resulting from vitamin depletion or mutations affecting mitochondrial function.

In SCA1, nuclear and cytosolic protein aggregates are associated with neurodegeneration of cerebellar Purkinje neurons. Although many protein interaction partners of ataxin1 have been identified and its impact on transcriptional dysregulation has been stated, the clear pathophysiologic processes leading to cell death remain to be elucidated [3]. Few hints point towards impeded antioxidant mechanisms such as reduced Cu/ZnSOD dismutase activity with subsequent accumulation of reactive oxygen species in the disease process [23]. Hypothetically the patchy pattern of temporal RNFLT reduction seen in our patients could be caused by localised breakdown of oxidative defense mechanisms.

In terms of retinal damage other previously examined ataxia disorders such as Friedreich's ataxia and neuropathy ataxia retinitis pigmentosa (NARP) belonging to the group of syndromic mitochondriopathies present with more general retinal thinning [24] [25]. One explanation could be the disruption of complex I, II and III as compared to only complex I in the non-syndromic mitochondrial neuropathies [22]. However, the role of respiratory chain complexes in SCA1 is not clear yet.

In SCA7, along with a peripheral RNFLT reduction even a sparing of the temporal sector has been observed in RNFLT measurements resembling the pattern in other retinal dystrophies such as retinitis pigmentosa [26]. Key to understanding the complementary findings could be the diverse function of ataxin 1 and ataxin7. In SCA7 a pronounced retinal cone-rod dystrophy associated with visual loss is a characteristic feature especially in early disease stages. Additionally in late stage disease a maculopathy can evolve (‘bull’s eye' maculopathy) [27]. In transgenic mice it has been shown that ataxin 7 can suppress a cone-rod homebox protein transactivation in retinal photoreceptor cells thus controlling expression level of several photoreceptor-specific genes such as rhodopsin [28]. The relative sparing of the temporal sector has been discussed as possible result of the relative sparing of the inner macula around the central fovea associated with normal ganglion cell layer thickness of the peripapillary temporal quadrant in earlier disease stage [6].

These distinct patterns raise the question whether OCT may serve a purpose in differential diagnosis in ataxia disorders, a topic yet to be addressed by examination of larger patient cohorts composed of different ataxia disorders in direct comparison. Additionally larger studies should allow more precise layer segmentation analysis. A longitudinal study design could also serve to evaluate potential use of RNFLT as non invasive disease progression marker.
